# Physiological Mechanisms Mediating the Coupling between Heart Period and Arterial Pressure in Response to Postural Changes in Humans

**DOI:** 10.3389/fphys.2017.00163

**Published:** 2017-03-27

**Authors:** Alessandro Silvani, Giovanna Calandra-Buonaura, Blair D. Johnson, Noud van Helmond, Giorgio Barletta, Anna G. Cecere, Michael J. Joyner, Pietro Cortelli

**Affiliations:** ^1^Department of Biomedical and Neuromotor Sciences, University of BolognaBologna, Italy; ^2^IRCCS Bologna Institute of Neurological SciencesBologna, Italy; ^3^Center for Research and Education in Special Environments, Department of Exercise and Nutrition Sciences, University at BuffaloBuffalo, NY, USA; ^4^Department of Anesthesiology, Mayo ClinicRochester, MN, USA

**Keywords:** baroreflex, heart, arterial blood pressure, central venous pressure, tilt table test, seated position, standing position, hemorrhage

## Abstract

The upright posture strengthens the coupling between heart period (HP) and systolic arterial pressure (SAP) consistently with a greater contribution of the arterial baroreflex to cardiac control, while paradoxically decreasing cardiac baroreflex sensitivity (cBRS). To investigate the physiological mechanisms that mediate the coupling between HP and SAP in response to different postures, we analyzed the cross-correlation functions between low-frequency HP and SAP fluctuations and estimated cBRS with the sequence technique in healthy male subjects during passive head-up tilt test (HUTT, *n* = 58), during supine wakefulness, supine slow-wave sleep (SWS), and in the seated and active standing positions (*n* = 8), and during progressive loss of 1 L blood (*n* = 8) to decrease central venous pressure in the supine position. HUTT, SWS, the seated, and the standing positions, but not blood loss, entailed significant increases in the positive correlation between HP and the previous SAP values, which is the expected result of arterial baroreflex control, compared with baseline recordings in the supine position during wakefulness. These increases were mirrored by increases in the low-frequency variability of SAP in each condition but SWS. cBRS decreased significantly during HUTT, in the seated and standing positions, and after blood loss compared with baseline during wakefulness. These decreases were mirrored by decreases in the RMSSD index, which reflects cardiac vagal modulation. These results support the view that the cBRS decrease associated with the upright posture is a byproduct of decreased cardiac vagal modulation, triggered by the arterial baroreflex in response to central hypovolemia. Conversely, the greater baroreflex contribution to cardiac control associated with upright posture may be explained, at least in part, by enhanced fluctuations of SAP, which elicit a more effective entrainment of HP fluctuations by the arterial baroreflex. These SAP fluctuations may result from enhanced fluctuations of vascular resistance specific to the upright posture, and not be driven by the accompanying central hypovolemia.

## Introduction

The upright posture challenges blood pressure and results in blood pooling and increased capillary ultrafiltration below the heart. The resultant decrease in central venous pressure (CVP) weakens cardiac contraction and leads to orthostatic hypotension if not compensated by the autonomic and skeletal muscle systems (Freeman et al., 2011). The arterial baroreflex modulates autonomic control of the heart and resistance vessels as a function of arterial wall tension, sensed by baroreceptors in the walls of the carotid sinuses and the aortic arch (Mancia and Mark, 2011). The arterial baroreflex competes for autonomic control of the heart and resistance vessels with other reflexes, such as the arterial chemoreflex, and with central autonomic commands (Silvani et al., 2016). Standing upright produces an exaggerated fall of arterial pressure in patients with bilateral carotid sinus denervation consequent to carotid body tumor resection, suggesting a key role of carotid baroreceptors in the hemodynamic compensation of upright posture (Smit et al., 2002). Accordingly, the analysis of spontaneous cardiovascular fluctuations, performed with a range of techniques designed to detect causality based on information theory, indicates that the upright posture increases the contribution of the arterial baroreflex to cardiac control (Nollo et al., 2005; Porta et al., 2011, 2014, 2015; Faes et al., 2013; Zamunér et al., 2015). Somewhat paradoxically, however, the cardiac baroreflex sensitivity (cBRS) estimated based on spontaneous fluctuations decreases in the upright position (Steptoe and Vögele, 1990; O'Leary et al., 2003; Nollo et al., 2005; Faes et al., 2013; Schwartz et al., 2013; Zamunér et al., 2015). The physiological mechanisms underlying these posture-related changes in cardiovascular coupling still remain unclear.

Potential insights may come from a different line of research, which investigated sleep-related differences in cardiovascular coupling with a simple linear technique, the cross-correlation function (CCF) between fluctuations of systolic arterial pressure (SAP) and heart period (HP). The CCF yields the linear correlation coefficient between HP and SAP as a function of the time shift between these variables. The sign of the time shift indicates whether HP fluctuations precede (positive sign) or are preceded by (negative sign) those of SAP. Previous experimental (Silvani et al., 2008, 2013) and theoretical (Silvani et al., 2011) research on human subjects indicates that the CCF between the LF fluctuations of HP and SAP may show a positive peak at negative time shifts. This peak indicates a pattern of positive correlation between the values of HP and the previous values of SAP, which is consistent with arterial baroreflex control of the heart (Silvani et al., 2011). The CCF analysis uncovered differences in cardiovascular coupling between non-rapid-eye-movement sleep (slow-wave sleep, SWS) and wakefulness in the lying position, consistent with a greater baroreflex contribution to cardiac control during SWS both in human subjects (Silvani et al., 2008, 2013) and in animal models (Silvani et al., 2005, 2010, 2012). The interpretation of these CCF patterns of cardiovascular coupling was supported and refined with an *in-silico* study (Silvani et al., 2011) based on an extensively validated non-linear model of the cardiovascular system (Ursino, 1998; Ursino and Magosso, 2000). This study suggested that the CCF pattern of positive correlation between SAP and the previous HP values, which is consistent with arterial baroreflex control of HP: (a) ensues as a result of baroreflex buffering of SAP fluctuations generated by changes of vascular resistance of whichever cause; (b) is dampened by decreases in the maximal sensitivity of the baroreflex, computed at the centering point of the baroreflex sigmoid function; (c) is dampened by central autonomic commands acting on the heart; and (d) is not explained by hypovolemia *per se* (Silvani et al., 2011). Application of the CCF analysis in previous work did not fully replicate the increased baroreflex contribution to cardiac control in the upright position, which was detected based on corrected conditional entropy, an information-domain analysis technique (Faes et al., 2013). It is, therefore, presently unclear whether the interpretative framework of CCF patterns (Silvani et al., 2011) may be extrapolated to the posture-related changes in cardiovascular coupling. If this were the case, since the CCF pattern of baroreflex cardiac control is dampened by reduced baroreflex sensitivity (Silvani et al., 2011), the increased baroreflex contribution to cardiac control in the upright position would appear at odds with the decrease in cBRS. However, this paradox may be explained based on evidence that the cBRS decrease in the upright position reflects reduced sensitivity at the baroreflex operating point, whereas the sensitivity at the baroreflex centering point does not change (Schwartz et al., 2013). These considerations raise the hypothesis that the cBRS decrease in the upright position is a byproduct of a decrease in the modulation of cardiac vagal activity, concomitant with the decrease in the mean HP values.

In the present study, we tested the hypotheses that in the upright position, the increased baroreflex contribution to cardiac control does not result from central hypovolemia, but rather reflects enhanced SAP fluctuations driven by vascular resistance, while the cBRS decrease reflects decreased cardiac vagal modulation. We went beyond the widely studied paradigm that compares supine wakefulness with passive standing (head-up tilt test, HUTT), including subjects lying asleep (SWS), seating, actively standing, and lying awake supine after controlled blood loss to cause central hypovolemia. We re-assessed the issue of whether the CCF analysis, a simple and widely available linear technique, is suitable to detect the increased baroreflex contribution to cardiac control in the upright position, which has been reported using sophisticated analysis techniques designed to detect causality based on information theory. Finally, we analyzed the links between the HP vs. SAP coupling, as assessed with the CCF analysis, and widely-applied indexes of cardiovascular variability in the time and frequency domains, including estimates of cBRS with the sequence technique (Bertinieri et al., 1988), by applying a data-driven hierarchical clustering approach.

## Materials and methods

### Ethical approval

This study involved the analysis of data from 74 human subjects. All subjects provided written informed consent to their research protocol, which conformed to the principles of the Declaration of Helsinki and received prior approval by the institutional review boards of the University of Bologna (approval code 876/CE) and the Mayo Clinic (approval code 11-3071).

### Subjects and recordings

#### Experiment 1: cardiovascular coupling during passive upright tilt

The analysis was performed on data obtained from 58 male subjects aged 38 ± 1 years (range 20–60 years), who were referred to the department of biomedical and neuromotor sciences (DIBINEM) and IRCCS Bologna institute of neurological sciences of the university of Bologna, Italy, for suspected neurally mediated syncope, but whose HUTT resulted negative. None of the subjects included in this experiment experienced symptoms of pre-syncope or syncope during the HUTT. The subjects self-reported to abstain from drugs and medications. Cardiac, endocrine, metabolic, and renal diseases were excluded on the basis of self-reported health history, physical examination, and routine laboratory tests. Before the beginning of the study, the subjects were instructed to abstain from heavy physical activity for 24 h and from alcohol and caffeinated beverages for 12 h. The subjects had a light breakfast before the recordings, which were performed in the morning. Values of RR interval (HP) and arterial pressure (Finometer Midi, Finapres Medical Systems, Amsterdam, The Netherlands) were recorded continuously with a polygraph amplifier (Model 15LT, Grass Technologies, Quincy, MA) that was connected to an analogic to digital converter operated by custom software (SparkBio Srl, Bologna, Italy). Recordings were performed with the subjects at baseline, lying awake in the supine position, then during HUTT at 65° for 10–30 min.

#### Experiment 2: cardiovascular coupling during active upright standing, while seated, and during slow-wave sleep

The analysis was performed on data from 8 healthy male subjects aged 39 ± 5 years, who were enrolled by the autonomic unit of the DIBINEM and IRCCS Bologna institute of neurological sciences of the university of Bologna, Italy, and were recorded with the same setup as for Experiment 1. The subjects self-reported to abstain from drugs and medications, had values of the apnea-hypopnea index <10, and did not complain of daytime sleepiness. Cardiac, endocrine, metabolic, and renal diseases were excluded on the basis of self-reported health history, physical examination, and routine laboratory tests. Before the beginning of the study, the subjects were instructed to abstain from heavy physical activity for 24 h and from alcohol and caffeinated beverages for 12 h. During the study, subjects lived in a room with a controlled temperature (24 ± 1°C), humidity (40–50%) and light–dark schedule (light off from 23:00 h to 07:00 h). Subjects were required to lie in bed, except for eating (1,800 kcal per day divided into 3 meals and 3 snacks with a fixed time schedule), and were allowed to read, watch television and sleep *ad libitum*. Continuous non-invasive recordings of physiological variables were performed for 48 h. Finger arterial pressure was measured with the volume-clamp method (Portapres Model-2; Finapres Medical Systems, Amsterdam, the Netherlands). Electrocardiogram, electroencephalogram (C3-A2 and C4-A1 leads), electrooculogram, electromyogram (submentalis muscle) and ventilation were recorded with a Colleague recorder (Albert Grass heritage, Model PSG16P-1; Astro-Med Inc., West Warwick, RI). The present study included recordings obtained in the morning while subjects were asked to lie awake in the supine position, then to sit in a chair, and finally to stand actively upright. None of the subjects experienced symptoms of pre-syncope or syncope during the protocol. This study also reported the analysis of recordings of these same subjects during nocturnal SWS (stages 3 and 4 of non-rapid-eye-movement sleep with Rechtschaffen and Kales classification on 30 s epochs), to allow for direct comparison with previous work (Silvani et al., 2013).

#### Experiment 3: cardiovascular coupling during progressive blood loss

We also analyzed data from a previously published dataset (Johnson et al., 2014) of 8 healthy male subjects aged 32 ± 3 years, who underwent stepwise reductions in circulating blood volume. Details of the experimental design and methods are reported elsewhere (Johnson et al., 2014). All subjects reported being free of cardiovascular, respiratory, neurologic, or metabolic diseases, were non-obese (body mass index <30) and non-smokers, and did not take any medications. At 07:00 h, following an overnight fast, subjects consumed a small breakfast (240 kcal) and drank 250 ml of water before being instrumented in the supine position in a temperature-controlled room (20–22°C). A 3-lead electrocardiogram was used to continuously record RR interval (HP). Three vascular catheters were introduced using aseptic techniques under local anesthesia (2% lidocaine) and ultrasound guidance. A 16-gauge catheter was introduced into an antecubital vein, connected to a high-resolution transducer (FloTrac; Edwards Lifesciences, Irvine, CA) and advanced until an appropriate CVP waveform was obtained, then positioned at heart level for continuous CVP measurement. A 14-gauge catheter was inserted in an antecubital vein to facilitate blood removal. A 20-gauge, 5-cm catheter was placed into a brachial artery, connected to a high-resolution transducer (FloTrac; Edwards Lifesciences) and positioned at heart level for continuous arterial pressure measurement. Subjects were instructed not to cross their legs or contract any muscles in their lower body throughout the protocol. At least 30 min following invasive instrumentation, recordings were performed during a 5 min baseline period with the subjects lying awake in the supine position. Then, 3 aliquots of 333 mL of blood were removed via gravity from an antecubital vein, and recordings were performed during the 5 min periods separating each aliquot. None of the subjects experienced symptoms of pre-syncope or syncope during the protocol.

### Data analysis

Data analysis was performed with routines written in Matlab (the Mathworks, Inc.) and with the same approach for all experiments. The analysis was based on the whole available beat-to-beat time series of HP, of SAP, of diastolic arterial pressure (DAP), and, in Experiment 3, also of CVP. The duration of these time series was 14 ± 1 and 17 ± 1 min in Experiment 1 with subjects in the supine and HUTT conditions, respectively; 14 ± 2, 5, 5, and 148 ± 16 min in Experiment 2 with subjects in the supine, seated, and active standing positions and during SWS, respectively; 5 min at baseline and after each step of blood loss in Experiment 3.

Artifacts due to measurement noise, e.g., because of occasional subject movements, or Finapres/Portapres calibrations, were automatically detected based on the magnitude of differences in HP, SAP, or CVP-values between subsequent beats, and the accuracy of determination was confirmed by visual check of the time series. The total duration of beats with artifacts in at least one signal, expressed as a percentage of the analyzed recording time, amounted to 4.5 ± 0.2 and 4.6 ± 0.2% in Experiment 1 with subjects in the supine and HUTT conditions, respectively; to 8.4 ± 1.2, 6.7 ± 0.6, 6.1 ± 0.5, and 5.1 ± 0.3% in Experiment 2 with subjects in the supine, seated, and active standing positions and during SWS, respectively; and to 0.3 ± 0.2, 1.0 ± 0.4, 0.7 ± 0.4, and 1.7 ± 0.7% at baseline and after each progressive step of blood loss in Experiment 3. The beats with artifacts were excluded from the computation of indexes based on beat-to-beat values in the time domain, whereas they were substituted by means of piecewise cubic spline interpolation for the computation of indexes based on the CCF analysis or on spectral analysis.

In particular, the following cardiovascular indexes were computed based on beat-to-beat values in the time domain: the mean values of SAP, HP, and CVP, the square root of the mean squared differences of the successive values of HP (RMSSD), and the cBRS. RMSSD estimates the short-term variability of HP values between successive heart beats (Task force of the european society of cardiology and the north american society of pacing and electrophysiology, 1996), reflecting the vagal modulation of HP (van den Berg et al., 1997). cBRS estimates the arterial baroreflex change in HP caused by a unit change in SAP, and was assessed with the sequence technique (Bertinieri et al., 1988) as previously described in detail (Silvani et al., 2008). Additional cardiovascular indexes were computed based on spectral analysis or on CCF analysis after resampling the beat-to-beat time series at 4 Hz by linear interpolation, and were averaged over consecutive data subsets of 5 min duration overlapped for 4 min (Silvani et al., 2008, 2013). Spectral analysis was performed with fast Fourier transforms on detrended and Hann-windowed data subsets of HP and SAP. The spectral powers of HP and SAP in the low-frequency (LF, 0.04-0.15 Hz; HP-LFSD and SAP-LFSD) and high-frequency (HF, 0.15-0.4 Hz; HP-HFSD and SAP-HFSD) bands were computed, square rooted, and scaled for expression in units of standard deviation. The CCF between HP and SAP was computed on detrended and low-pass filtered (<0.15 Hz, 10-pole Butterworth filter; phase distortion avoided by forward and reverse filtering) data subsets at time shifts between −25 and 25 s, and normalized so that the autocorrelations at 0 time shift were identically 1 (Silvani et al., 2008, 2013). The CCF between the LF fluctuations of HP and SAP in humans may show a positive peak at negative time shifts between 0 and −10 s (Silvani et al., 2008, 2011, 2013). This peak indicates a pattern of positive correlation between the values of HP and the previous values of SAP, which is consistent with arterial baroreflex control of the heart (Silvani et al., 2011). On this basis, the index pCC in this study was computed as the maximum value of the CCF at time shifts between −10 and 0 s.

### Statistical analysis

Statistical tests were performed with the SPSS software (SPSS, Inc.) by means of analysis of variance (ANOVA) or covariance (ANCOVA) with general linear model procedures and Huyn-Feldt correction for lack of the sphericity assumption, and of *t*-tests. In Experiment 1, the discrimination of the supine vs. HUTT condition of individual subjects was assessed with a receiver operating characteristic (ROC) function, which plotted detection sensitivity vs. false positive rate (i.e., 1–specificity). In the same experiment, a data-driven, exploratory approach was applied to map the links between the different cardiovascular indexes computed, employing a hierarchical cluster analysis based on squared Euclidean distances between z-scores within variables. The hypotheses generated by the cluster analysis (i.e., of links between SAP-LFSD and pCC, and between RMSSD and cBRS) and were then evaluated by testing for significant Pearson's linear correlation between changes in these indexes on passing from the supine position to HUTT. In these analyses, subjects were classified as responders if pCC increased from the supine position to HUTT, and as non-responders otherwise. Based on the results of these analyses, and in order to limit the number of statistical comparisons performed, the analysis of Experiments 2 and 3 focused on SAP-LFSD, pCC, RMSSD, and cBRS. Statistical significance for all tests was set at *P* < 0.05. Data are reported as mean ± SEM.

## Results

### Experiment 1: cardiovascular coupling during passive upright tilt

HUTT caused significant increases of the mean value of DAP and SAP and of the variability of SAP in the LF (SAP-LFSD) and the HF (SAP-HFSD) ranges (Table 1) above the respective baseline values in the supine position. Conversely, HUTT entailed significant decreases in the mean value of HP, in the variability of HP either between successive heart beats (RMSSD index) or in the HF frequency range (HP-HFSD), and in cBRS compared to the supine position. The variability of HP in the LF range (HP-HFSD) did not change significantly during HUTT compared to the supine position.

**Table 1:** **Mean values and variability of arterial pressure and heart period during 65° head-up tilt test**.

	**SUPINE**	**HUTT**
DAP (mm Hg)	62.2 ± 1.0	74.0 ± 1.2^*^
SAP (mm Hg)	116.1 ± 1.4	126.1 ± 1.5^*^
SAP-LFSD (mm Hg)	2.6 ± 0.1	4.1 ± 0.2^*^
SAP-HFSD (mm Hg)	0.9 ± 0.0	1.9 ± 0.1^*^
HP (ms)	954.0 ± 15.8	768.6 ± 12.6^*^
HP-LFSD (ms)	27.6 ± 1.6	27.4 ± 1.3
HP-HFSD (ms)	19.4 ± 1.3	10.6 ± 0.7^*^
RMSSD (ms)	39.6 ± 2.9	19.9 ± 1.1^*^
cBRS (ms mm Hg^−1^)	15.8 ± 0.9	6.4 ± 0.4^*^

*HUTT, 65° head-up tilt test. SUPINE, supine position before HUTT. DAP and SAP, diastolic and systolic arterial pressure, respectively. HP, heart period. SAP-LFSD, SAP-HFSD, HP-LFSD, HP-HFSD: square root of the low-frequency (LF, 0.04–0.15 Hz) or high-frequency (HF, 0.15–0.4 Hz) spectral powers of SAP or HP, respectively, scaled for expression in units of standard deviation. RMSSD, root mean square of the differences between consecutive values of HP. cBRS, spontaneous cardiac baroreflex sensitivity (sequence technique). Data are shown as mean ± SEM in 58 healthy male controls*.

* *P < 0.05 vs. SUPINE (t-test)*.

**Figure 1:**
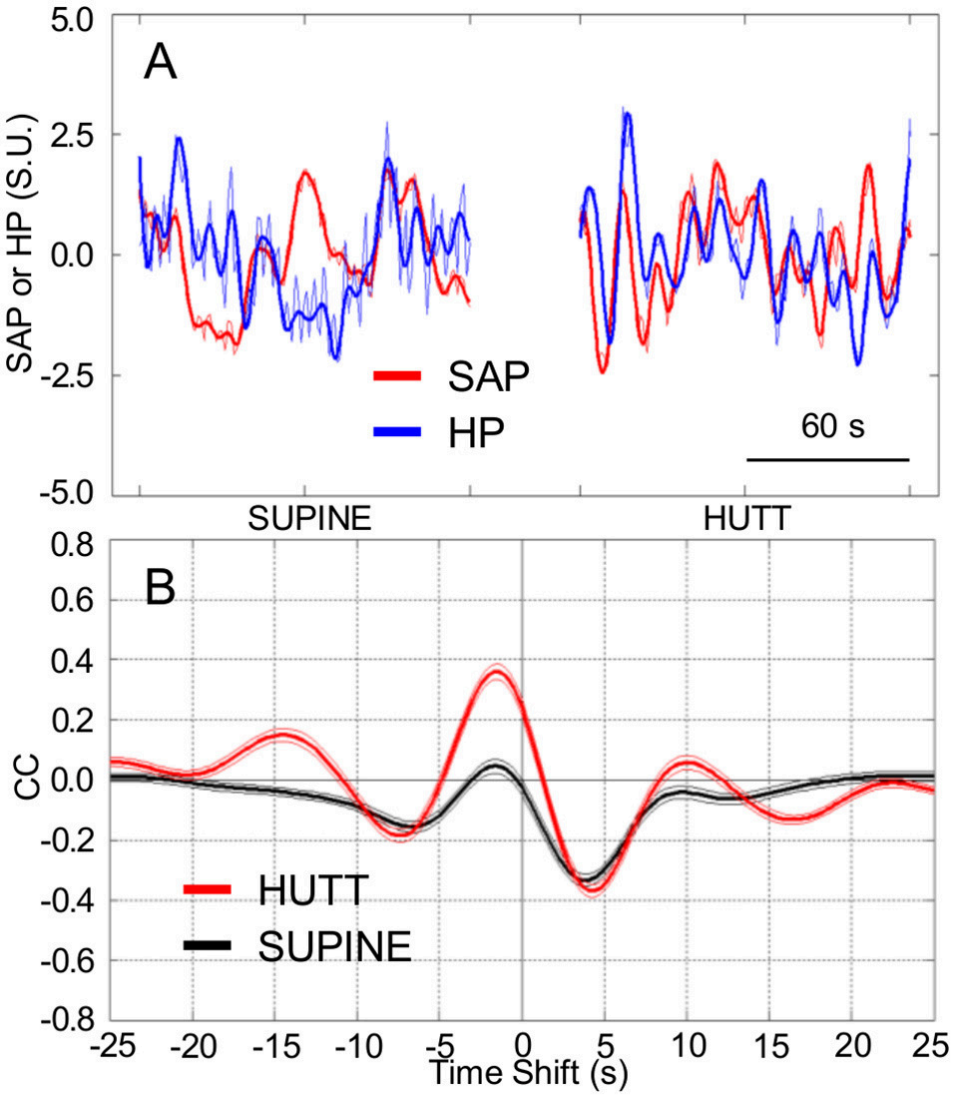
**Cardiovascular coupling during 65° head-up tilt test**. HUTT, 65° head-up tilt test. SUPINE, supine position before HUTT. HP, heart period. SAP, systolic arterial pressure. CCF, cross-correlation functions between low-pass filtered (<0.15 Hz) spontaneous fluctuations of SAP and HP. **(A)** Representative time series of HP and SAP. Thick lines indicate the result of low-pass filtering <0.15 Hz. Values are in standardized units (S.U.). **(B)** CCF, with negative time shifts indicating that HP followed SAP. Data are shown as mean ± SEM in 58 male subjects.

The values of HP fluctuated in parallel with the previous values of SAP to a greater extent during HUTT than in the supine position (Figure 1A). The pattern of cardiovascular coupling that prevailed during HUTT was thus such that HP tended to increase following increases in SAP and to decrease following decreases in SAP, consistently with the arterial baroreflex control of HP (Silvani et al., 2011). These observations were quantified by analyzing the CCF between the LF fluctuations of HP and those of SAP. Only during HUTT did this CCF show a positive peak at negative time shifts, i.e., for HP fluctuations that followed those of SAP (Figure 1B). Accordingly, the value of pCC, which quantify the height of the CCF peak at negative time shifts, were significantly higher during HUTT (0.39 ± 0.02) than in the supine position (0.08 ± 0.02; *P* < 0.001, *t*-test).

**Figure 2:**
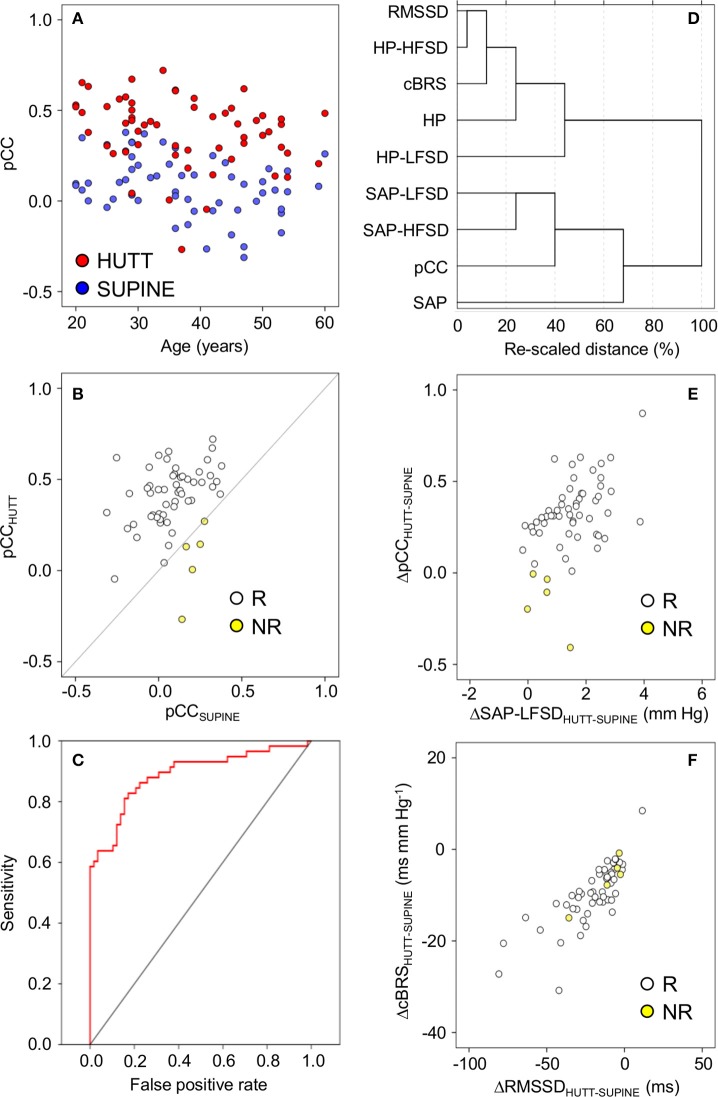
**Relationships between indexes of cardiovascular coupling and cardiovascular variability during 65° head-up tilt test**. HUTT, 65° head-up tilt test. SUPINE, supine position before HUTT. HP, heart period. SAP, systolic arterial pressure. CCF, cross-correlation functions between low-pass filtered (<0.15 Hz) time series of HP and SAP. pCC, correlation coefficient at the CCF peak at time shifts between–10 and 0 s. SAP-LFSD, SAP-HFSD, HP-LFSD, HP-HFSD: square root of the low-frequency (LF, 0.04–0.15 Hz) or high-frequency (HF, 0.15–0.4 Hz) spectral powers of SAP or HP, respectively, scaled to units of standard deviation. RMSSD, root mean square of differences between consecutive values of HP. cBRS, spontaneous cardiac baroreflex sensitivity (sequence technique). **(A)** Values of pCC as a function of subject age. **(B)** Values of pCC in responder (R) or non-responder (NR) subjects, in whom pCC increased or did not increase from SUPINE to HUTT, respectively. **(C)** Receiver-operating characteristic (ROC) curve of the discrimination between SUPINE and HUTT based on pCC. **(D)** Dendrogram of the hierarchical cluster analysis of the variables listed in the y-axis, measured during SUPINE and HUTT, and scaled by the joining distances of the clusters. **(E,F)** Differences (Δ) between HUTT and SUPINE in pCC and SAP-LFSD and in cBRS and RMSSD, respectively. In **(A,B,E,F)** each circle indicates values in a single subject. Data are shown as mean ± SEM in 58 male subjects.

The increase in pCC from the supine position to HUTT was significant (ANCOVA: *P* = 0.006) irrespective of the age of the subjects (ANCOVA: *P* = 0.914 for the interaction between age and body position), although the values of pCC in the supine position as well as during HUTT were lower in older subjects (ANCOVA: *P* = 0.005 for the effect of age; Figure 2A). pCC increased from the supine position to HUTT in most subjects (91.4%, or 53 out of 58 subjects, hereafter defined as the responders, in contrast with the remaining 5 subjects, defined as non-responders; Figure 2B). The robustness of the changes in pCC associated with HUTT was supported by a ROC function analysis, which demonstrated that pCC significantly (*P* < 0.001) and effectively (AUC = 88.9 ± 3.1%) discriminated whether recordings were performed during HUTT or in the supine position (Figure 2C).

A hierarchical cluster analysis of these results indicated that pCC and cBRS belonged to two different clusters of variables, which were more closely related to the values of SAP and HP, respectively (Figure 2D). In particular, pCC was closely related to a sub-cluster of variables including SAP-LFSD and SAP-HFSD, whereas cBRS was closely related to another sub-cluster including RMSSD and HP-HFSD. To simplify interpretation of results, in follow-up analyses, we focused on SAP-LFSD, which was computed based on LF fluctuations as was pCC, as representative of one sub-cluster, and on RMSSD, which was computed in the time-domain as was cBRS, as representative of the other sub-cluster.

Supporting the heuristic value of this exploratory cluster analysis, we found that the increase in pCC from the supine position to HUTT was positively and significantly correlated with the increase in SAP-LFSD in responders (Pearson's *r* = 0.44, *P* = 0.001, implicating *r*^2^ = 19% of shared variance; Figure 2E). The differences in pCC and SAP-LFSD between the supine position and HUTT were both significantly larger in responders (pCC: +0.34 ± 0.02; SAP-LFSD: +1.6 ± 0.1 mm Hg) than in non-responders (pCC: −0.15 ± 0.07, *P* < 0.001; SAP-LFSD: +0.6 ± 0.3 mm Hg, *P* = 0.025, *t*-test). On the other hand, neither the decrease in cBRS (*P* = 0.296, *t*-test) nor that in RMSSD (*P* = 0.331, *t*-test) from the supine position to HUTT differed significantly between non-responders and responders. In responders, the decrease in cBRS from the supine position to HUTT was positively and significantly correlated with the decrease in RMSSD (Pearson's *r* = 0.79, *P* < 0.001, implicating *r*^2^ = 63% of shared variance; Figure 2F).

### Experiment 2: cardiovascular coupling during active upright standing, while seated, and during slow-wave sleep

**Figure 3:**
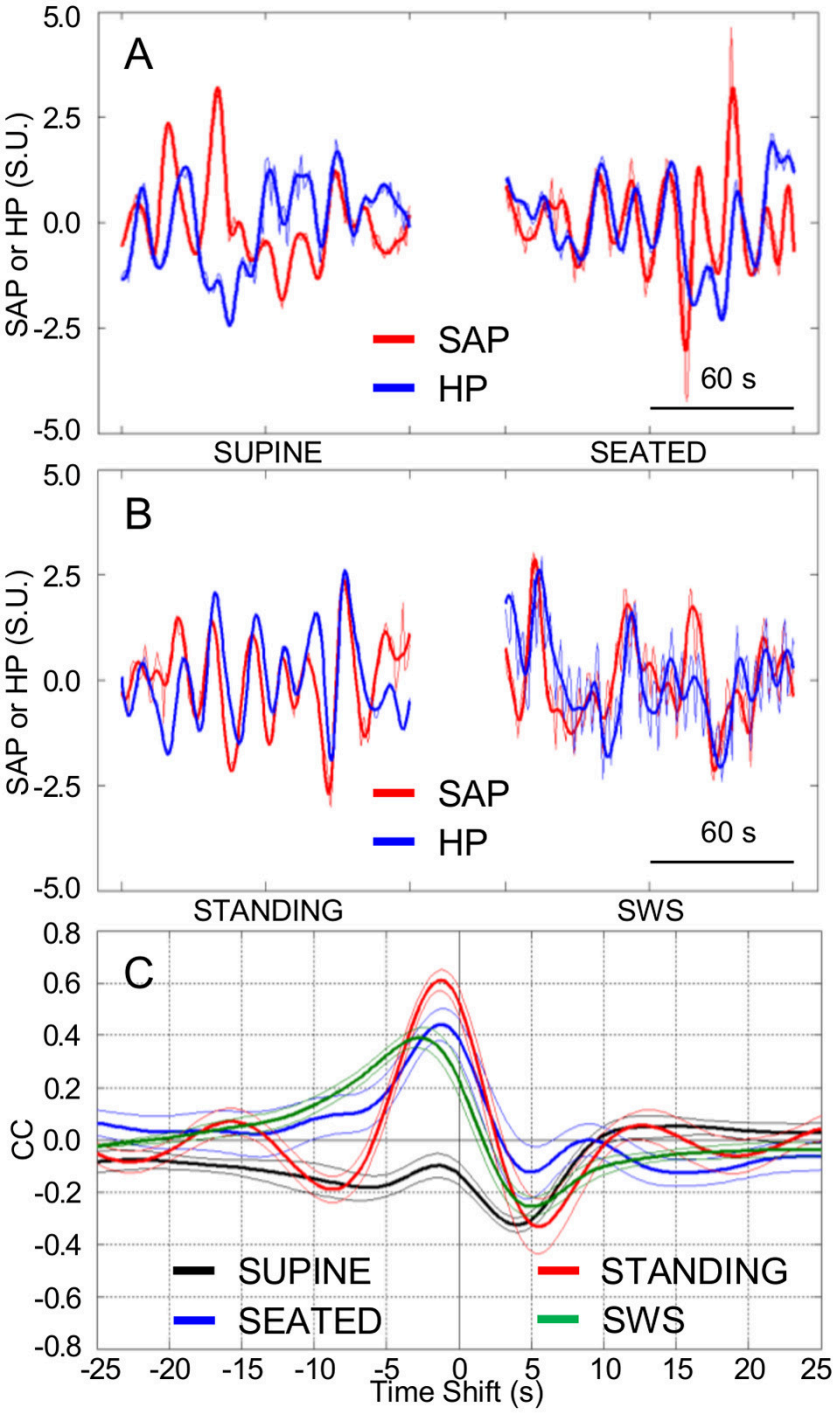
**Cardiovascular coupling during active upright standing, in the seated position, and during slow-wave sleep**. SEATED, seated position in a chair; SUPINE, supine position during wakefulness before sitting; STANDING, actively standing upright from the seated position; SWS, slow-wave sleep during the night. HP, heart period. SAP, systolic arterial pressure. CCF, cross-correlation functions between low-pass filtered (<0.15 Hz) spontaneous fluctuations of SAP and HP. **(A,B)** Representative time series of HP and SAP. Thick lines indicate the result of low-pass filtering <0.15 Hz. Values are in standardized units (S.U.). **(C)** CCF, with negative time shifts indicating that HP followed SAP. Data are shown as mean ± SEM in 8 healthy male subjects.

In line with the results of Experiment 1, HP fluctuations tended to be parallel with the previous SAP fluctuations to a greater extent while the subjects were seated (Figure 3A) and, even more so, while they were actively standing upright (Figure 3B), than while they rested awake in the supine position. Accordingly, the CCF between the LF fluctuations of HP and SAP in subjects seated or actively standing upright showed a clear-cut positive peak at negative time shifts, i.e., reflecting HP fluctuations that followed those of SAP. This pattern was similar to that occurring during SWS in the same subjects (Figure 3C).

**Figure 4:**
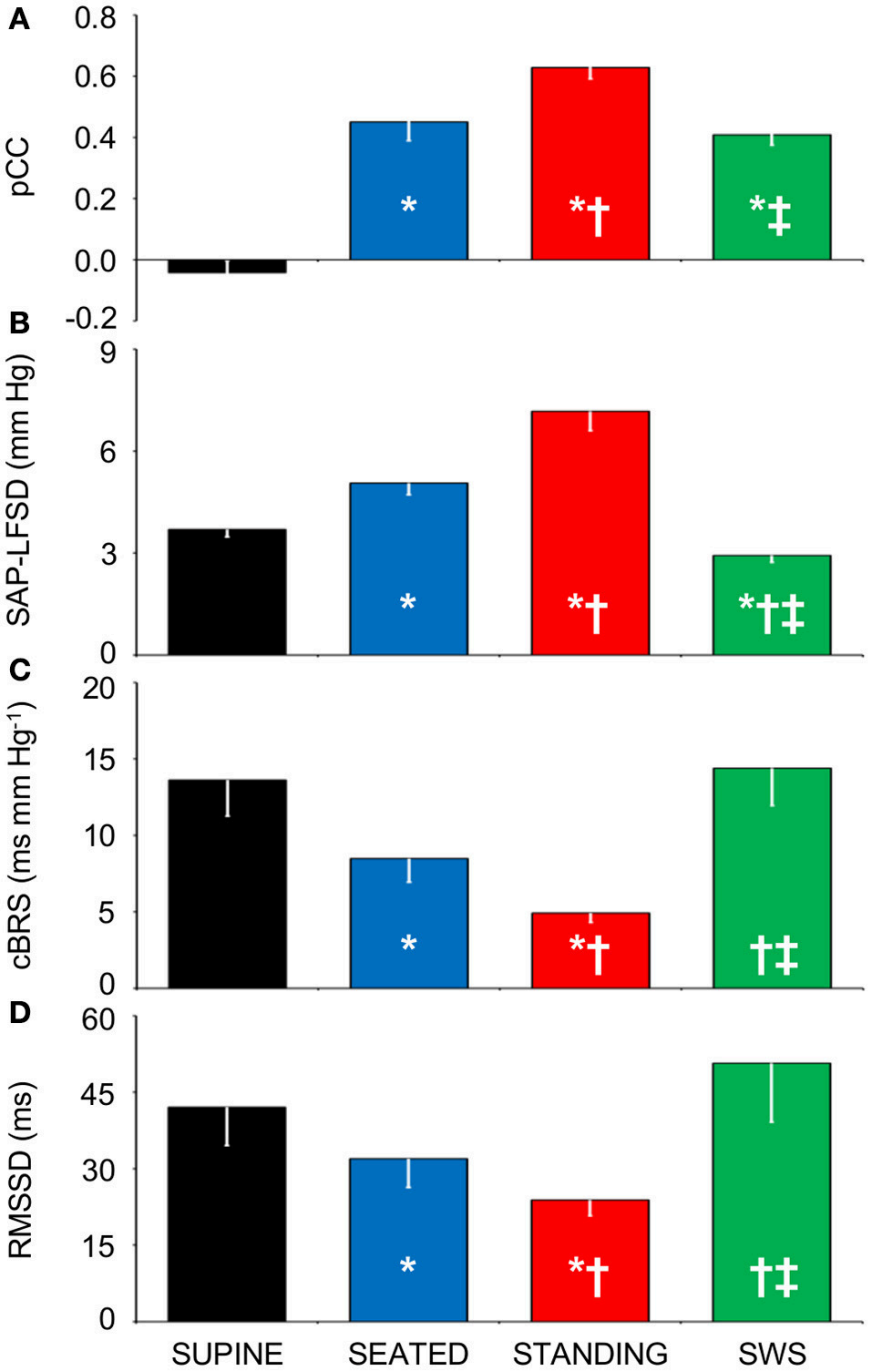
**Relationships between indexes of cardiovascular coupling and cardiovascular variability during active upright standing, in the seated position, and during slow-wave sleep. (A)** pCC, correlation coefficient at the peak of the cross-correlation functions between low-pass filtered (<0.15 Hz) fluctuations of heart period (HP) and systolic arterial pressure (SAP), at time shifts between −10 and 0 s. **(B)** SAP-LFSD, square root of the low-frequency (LF, 0.04–0.15 Hz) spectral power of SAP, scaled for expression in units of standard deviation. **(C)** cBRS, spontaneous cardiac baroreflex sensitivity. **(D)** RMSSD, root mean square of differences between consecutive values of HP. Data are shown as mean ± SEM in 8 healthy male subjects. ANOVA: *P* < 0.001 for pCC, SAP-LFSD, and cBRS; *P* = 0.013 for RMSSD. ^*^, ^†^, and ^‡^: *P* < 0.05 vs. SUPINE, vs. SEATED, and vs. STANDING, respectively (*t*-test).

Again supporting the heuristic value of the cluster analysis in Experiment 1, the values of pCC and those of SAP-LFSD computed in Experiment 2 progressively and significantly increased from the condition of lying awake supine to the seated position, and even more so to the active standing position (Figures 4A,B). Moreover, the values of cBRS and RMSSD (Figures 4C,D) progressively and significantly decreased during wakefulness from the supine to the seated and active standing positions. The mean values of HP also decreased during wakefulness from the supine to the seated and active standing positions. The mean values of DAP showed opposing changes, increasing during wakefulness from the supine to the seated and active standing positions, whereas the mean values of SAP did not differ significantly among these conditions (Table 2).

**Table 2:** **Mean values of arterial pressure and heart period during active upright standing, while seated, and during slow-wave sleep**.

	**SUPINE**	**SEATED**	**STANDING**	**SWS**
DAP (mm Hg)	70.8 ± 3.0	76.8 ± 2.8^*^	80.8 ± 3.1^*^^†^	59.9 ± 2.6^*^^†^^‡^
SAP (mm Hg)	134.5 ± 6.4	141.4 ± 5.0	142.7 ± 5.5	113.7 ± 5.8^*^^†^^‡^
HP (ms)	940.9 ± 51.6	847.4 ± 43.7^*^	745.2 ± 42.8^*^^†^	1068.5 ± 48.3^*^^†^^‡^

*SEATED, seated position in a chair; SUPINE, lying supine awake before assuming the seated position; STANDING, actively standing upright from the seated position; SWS, slow-wave sleep during the night. HP, heart period. DAP and SAP, diastolic and systolic arterial pressure, respectively. Data are shown as mean ± SEM in 8 healthy male subjects. ANOVA: P < 0.001 for DAP, SAP and HP*.

*, †, and ‡ *: P < 0.05 vs. SUPINE, vs. SEATED, and vs. STANDING, respectively (t-test)*.

During SWS, the values of pCC did not differ significantly from those in the seated position, and were intermediate between those in the supine position during wakefulness and those in the active standing position (Figure 4A). The values of SAP-LFSD (Figure 4B) and the mean values of DAP and SAP (Table 2) during SWS were significantly lower than in each other condition during wakefulness. The mean value of HP during SWS was instead significantly higher than in each other condition (Table 2). The values of cBRS and RMSSD (Figures 4C,D) during SWS did not differ significantly from those in subjects lying awake supine, and were significantly higher than those in the seated and active standing positions, once again in line with the heuristic derived from cluster analysis in Experiment 1.

### Experiment 3: cardiovascular coupling during progressive blood loss

In comparison with baseline conditions of subjects lying awake supine, the loss of 0.33 L blood significantly decreased the mean value of CVP without entailing significant changes in the mean values of SAP and HP (Table 3). Greater blood losses elicited further reductions in the mean values of CVP, with concomitant reductions in the mean values of HP and SAP. The mean values of DAP did not vary with blood loss.

**Table 3:** **Mean values of arterial pressure, heart period, and central venous pressure during progressive blood loss**.

	**BASELINE**	**BL 0.33 L**	**BL 0.67 L**	**BL 1 L**
DAP (mm Hg)	70.1 ± 1.9	70.0 ± 1.6	70.5 ± 1.9	70.1 ± 1.8
SAP (mm Hg)	133.9 ± 5.0	132.4 ± 4.5	129.0 ± 5.4^*^	124.9 ± 5.6^*^
HP (ms)	1041.0 ± 61.4	1005.3 ± 46.8	962.5 ± 43.7^*^^†^	906.9 ± 45.1^*^^†^^‡^
CVP (mm Hg)	8.1 ± 0.8	5.6 ± 0.8^*^	3.9 ± 1.0^*^^†^	2.5 ± 1.1^*^^†^^‡^

*BASELINE, baseline recordings in subjects lying awake supine before blood loss; BL 0.33 L, BL 0.67 L, and BL 1 L, recordings after a cumulative blood loss of 0.33 L, 0.67 L, and 1 L, respectively. HP, heart period. DAP and SAP, diastolic and systolic arterial pressure, respectively. CVP, central venous pressure. Data are shown as mean ± SEM in 8 healthy male subjects. ANOVA: P = 0.877 for DAP, P = 0.051 for SAP, P < 0.001 for HP and CVP*.

*, †, and ‡ *: P < 0.05 vs. BASELINE, vs. BL 0.33 L, and vs. BL 0.67 L, respectively (t-test)*.

**Figure 5:**
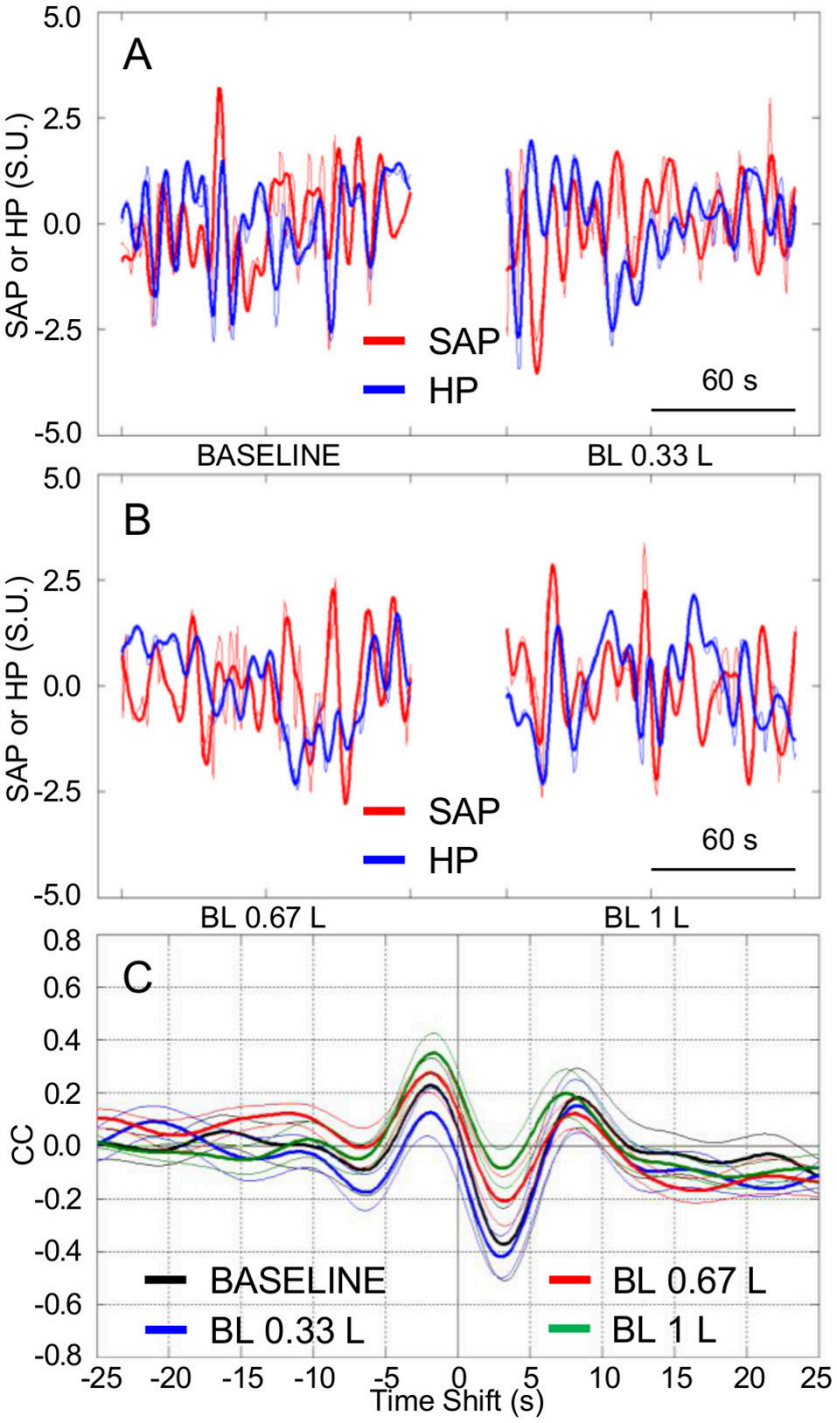
**Cardiovascular coupling during progressive blood loss in subjects lying awake supine**. BASELINE, baseline recordings in subjects lying awake supine before blood loss; BL 0.33 L, BL 0.67 L, and BL 1 L, recordings after a cumulative blood loss of 0.33 L, 0.67 L, and 1 L, respectively. HP, heart period. SAP, systolic arterial pressure. CCF, cross-correlation functions between low-pass filtered (<0.15 Hz) spontaneous fluctuations of SAP and HP. **(A,B)** Representative time series of HP and SAP. Thick lines indicate the result of low-pass filtering <0.15 Hz. Values are in standardized units (S.U.). **(C)** CCF, with negative time shifts indicating that HP followed SAP. Data are shown as mean ± SEM in 8 healthy male subjects.

**Figure 6:**
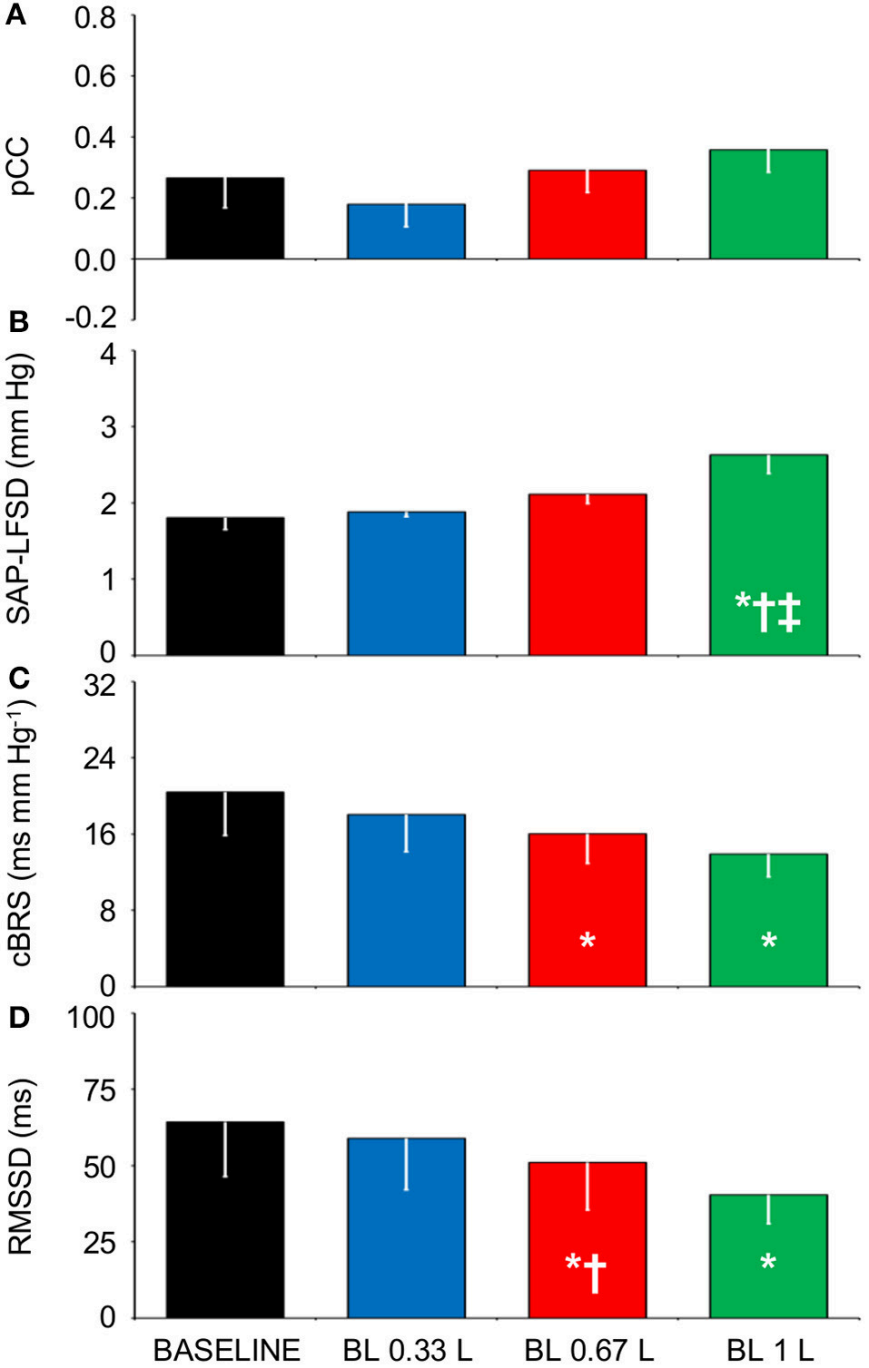
**Relationships between indexes of cardiovascular coupling and cardiovascular variability during progressive blood loss**. BASELINE, baseline recordings in subjects lying awake supine before blood loss; BL 0.33 L, BL 0.67 L, and BL 1 L, recordings after a cumulative blood loss of 0.33 L, 0.67 L, and 1 L, respectively. **(A)** pCC, correlation coefficient at the peak of the cross-correlation functions between low-pass filtered (<0.15 Hz) spontaneous fluctuations of heart period (HP) and systolic arterial pressure (SAP), at time shifts between −10 and 0 s. **(B)** SAP-LFSD, square root of the low-frequency (LF, 0.04–0.15 Hz) spectral power of SAP, scaled for expression in units of standard deviation. **(C)** cBRS, spontaneous cardiac baroreflex sensitivity. **(D)** RMSSD, root mean square of differences between consecutive values of HP. Data are shown as mean ± SEM in 8 healthy male subjects. ANOVA: *P* = 0.351 for pCC; *P* = 0.001 for SAP-LFSD; *P* = 0.032 for cBRS; *P* = 0.021 for RMSSD. ^*^, ^†^, and ^‡^: *P* < 0.05 vs. BASELINE, vs. BL 0.33 L, and vs. BL 0.67 L, respectively (*t*-test).

The tendency of HP fluctuations to be parallel with the previous values of SAP, which was observed in Experiment 1 during HUTT and in Experiment 2 in the seated and standing positions and during SWS, did not appreciably vary after progressive blood loss in the supine position (Figures 5A,B). Accordingly, the CCF between the LF fluctuations of HP and SAP showed a positive peak at negative time shift, which did not differ significantly in height after progressive blood loss (Figures 5C, 6A). The values of SAP-LFSD increased significantly above baseline conditions only after the loss of 1 L of blood (Figure 6B). This increase in SAP-LFSD was, however, quite limited (+0.8 ± 0.2 mm Hg) and similar in magnitude to the increase in SAP-LFSD during HUTT in the non-responder subjects of Experiment 1 (+0.6 ± 0.3 mm Hg, cf. above). The values of cBRS and RMSSD (Figures 6C,D) did not change significantly after a blood loss of 0.33 L, whereas they decreased significantly below baseline values after greater blood losses.

## Discussion

### Summary of the main findings

In this study, we re-assessed the issue of whether the CCF analysis is suitable to detect the increased baroreflex contribution to cardiac control in the upright position. Our results supported this view: with the CCF analysis, a relatively simple time-domain technique, we found that a range of passive (HUTT) and active (seated and standing positions) upright postures robustly entailed a positive correlation between HP and the previous values of SAP, which is consistent with arterial baroreflex control of HP, compared to the supine position. We then tested the hypotheses that in the upright position, the increased baroreflex contribution to cardiac control does not result from central hypovolemia, but rather reflects enhanced SAP fluctuations driven by fluctuations of vascular resistance, while the cBRS decrease reflects decreased cardiac vagal modulation. Our results fully supported these hypotheses. Accordingly, reductions in CVP in the supine position elicited by progressive blood loss did not significantly affect the positive correlation between HP and the previous values of SAP. Increases in the positive correlation between HP and the previous values of SAP were associated with increases in the LF variability of SAP during HUTT and in the seated and active standing positions, but not during SWS. The values of cBRS decreased in parallel with values of the RMSSD index during HUTT, in the seated and active standing positions, and after blood loss, compared to the supine position either during wakefulness or during SWS.

### The CCF analysis is sensitive to posture-related changes in cardiovascular coupling

A set of analysis techniques based on information theory (Nollo et al., 2005; Porta et al., 2011, 2014, 2015; Faes et al., 2013; Zamunér et al., 2015) has been applied to infer the direction and strength of causal links between the time series of SAP and HP. A rather straightforward interpretation of causality is afforded by these techniques at the expense of significant computational and theoretical complexity. Different techniques, such as a linear model-based approach and non-linear model-free analyses of local predictability and conditional entropy, may entail different levels of sensitivity and specificity to causal links (Faes et al., 2013; Porta et al., 2014). On the other hand, the computational and theoretical complexity of the CCF analysis is much more limited, and its similarity to the popular Pearson's correlation coefficient may make it widely accessible. However, the CCF analysis requires cautious inference of causality from correlation coefficients and time shifts based on physiological knowledge (Silvani et al., 2005, 2008, 2011). In particular, a positive correlation between HP and the previous values of SAP, i.e., a positive value of pCC, is consistent with the arterial baroreflex control of HP, which operates as a delayed negative feedback control, even if the time shift corresponding at the CCF peak overestimates the cardiac baroreflex latency. On the other hand, non-baroreflex autonomic control of the heart, including central autonomic commands on the heart and resistance vessels, entail episodes of cardiac acceleration and increase in arterial pressure, implying negative values of pCC, and a further slowing the apparent baroreflex dynamics as reflected by the time shift at the CCF peak. Thus, the CCF analysis does not provide independent information on central autonomic commands and the baroreflex, but rather lumped information on the relative contribution of each mechanism to cardiac control. The consistency of this interpretation of the CCF analysis is supported by mathematical modeling of human cardiovascular coupling (Silvani et al., 2011). In spite of its limitations, the present results demonstrate that the CCF analysis shows excellent sensitivity to the posture-related increase in the contribution of the arterial baroreflex to cardiac control: this increase was detected in 91.4% of subjects during HUTT, significantly discriminated HUTT from the lying supine position (Figure 2), and occurred in a graded fashion from the lying supine position to the seated and active standing positions (Figure 4), which are known to elicit different cardiovascular responses (Eckberg, 1980). Thus, our results indicate that the CCF analysis may represent a sensitive and widely-available complement to information-domain techniques in the assessment of posture-related changes in cardiovascular coupling.

In previous work, the enhancement of the baroreflex contribution to the coupling between HP and SAP during early HUTT could be detected both in control subjects and in subjects with neurally mediated syncope by employing the conditional entropy analysis, an information-domain analysis of causality, but only in control subjects by employing the CCF analysis (Faes et al., 2013). Taken together, our results and those of previous work (Faes et al., 2013) thus raise the hypothesis that the CCF analysis during HUTT may discriminate patients with neurally mediated syncope from control subjects. However, in a minority of subjects (5/58, the non-responders), we found that pCC did not increase during HUTT, yet no symptom of pre-syncope or syncope was experienced (Figure 2B). Since the subjects included in Experiment 1 were referred for evaluation because of suspected neurally mediated syncope (cf. Methods), we cannot exclude that at least some of them had a non-physiological response to upright posture. Moreover, our subdivision in responders and non-responders should be taken cautiously, as it was based on relatively minor changes of a single index (pCC). With these caveats in mind, our results suggest that the enhancement of the arterial baroreflex control of HP during HUTT is physiological, but is not necessary for orthostatic tolerance. Accordingly, there is evidence of individual variability in the recruitment of different and partially redundant baroreflex responses, such as increases of heart rate and burst incidence of muscle sympathetic nerve activity during upright standing (Burke et al., 1977). Moreover, neither fixed-rate cardiac pacing (Taylor and Eckberg, 1996) nor pharmacological cardiac vagal blockade (Ogoh et al., 2006) abolishes arterial pressure regulation during HUTT in healthy human subjects.

Analysis of information-domain causality has yielded contrasting results on the effects of subject age on the baroreflex contribution to cardiac control, which depended on the body position and the specific analysis technique employed (Porta et al., 2014). In particular, a linear model-based approach and a model-free analysis of conditional entropy did not detect significant age-related changes in the causality from SAP to HP, which is attributable to the arterial baroreflex, in subjects either lying supine or actively standing. Conversely, a model-free analysis of local predictability indicated a decrease in the causality from SAP to HP with age during active standing but not during supine rest (Porta et al., 2014). The enhancement of cardiac baroreflex control during HUTT detected by the CCF analysis was also robust to differences in subject age in our sample of subjects aged 20–60 years (Figure 2A). Nonetheless, we found that pCC decreased with subject age both in the supine position and during HUTT. With the caution that is mandatory in interpreting cross-sectional data, these results suggest that the control of HP in awake subjects lying supine shifts from a prevalence of the arterial baroreflex (positive pCC) to a prevalence of central autonomic commands (negative pCC) with increasing age.

### cBRS may decrease in the upright position as a byproduct of decreased modulation of cardiac vagal activity triggered by arterial baroreflex activation

Our finding that cBRS decreased with active and passive standing postures compared to the lying position (Table 1, Figure 4C) agrees with previous work (Steptoe and Vögele, 1990; O'Leary et al., 2003; Nollo et al., 2005; Faes et al., 2013; Schwartz et al., 2013; Zamunér et al., 2015). Moreover, our finding that cBRS in the supine position significantly decreased after the loss of more than 0.33 L of blood is in line with previous reports (Hughson et al., 1994; Saitoh et al., 2008) obtained using lower-body negative pressure, which also decreases CVP in the supine position. Previous work has shown that the cardiac baroreflex control during HUTT is reset toward higher values of arterial pressure and lower values of HP, but that its maximal sensitivity, corresponding to the centering point of the baroreflex sigmoid function, does not change (Schwartz et al., 2013). However, cardiac baroreflex sensitivity at the baroreflex operating point decreases during HUTT, and is accurately tracked by estimates of spontaneous cBRS (Schwartz et al., 2013). This raises the hypothesis that the cBRS decrease during HUTT may be a byproduct of a decrease in the modulation of cardiac vagal activity, concomitant with the decrease in the mean HP values. Our findings that decreases in cBRS during HUTT (Table 1, Figures 2D–F), in the seated and active standing positions (Figures 4C,D), and during progressive blood loss in the supine position (Figures 6C,D) were accompanied by corresponding decreases in RMSSD, which reflects the modulation of cardiac vagal activity (van den Berg et al., 1997), support this hypothesis.

### The upright posture specifically enhances SAP fluctuations

Theoretical (deBoer et al., 1987) and experimental (Bertram et al., 1998) evidence indicates that the LF fluctuations of vascular resistance are paradoxically enhanced by the arterial baroreflex control of sympathetic nerve activity to blood vessels, which resonates in the LF range due to delays in its effector response. We found that SAP-LFSD significantly increased during HUTT (Table 1, Figure 2E) and also in the seated and active standing positions (Figure 4B) compared to baseline recordings in the supine position, in line with a previous reports (Veerman et al., 1994; Barnett et al., 1999; Cooke et al., 1999; Marchi et al., 2016). On the other hand, we found that SAP-LFSD increased more modestly if at all in the supine position after blood loss (Figure 6B). These data may reflect a type II error due to sample size (*n* = 8), which was relatively small, albeit considerable in absolute terms with respect to the demanding experimental protocol of Experiment 3. Nonetheless, our data are in line with previous data that employed lower-body negative pressure to decrease CVP in the supine position (Kiviniemi et al., 2011; Aletti et al., 2012). Activation of the cardiopulmonary baroreflex may enhance the arterial baroreflex control of vascular resistance in human subjects (Victor and Mark, 1985). This is of relevance because the decrease in mean HP and mean SAP elicited by the loss of 0.67 L or 1 L blood in this study (Table 3) suggest activation of the arterial baroreflex in addition to cardiopulmonary reflex receptors (Mark and Mancia, 2011). Milder hypovolemia may also unload arterial baroreceptors, causing transient decreases in SAP that are rapidly buffered by the arterial baroreflex (Fu et al., 2009). We cannot directly compare the degree of central hypovolemia caused by progressive blood loss with that caused by HUTT, seating, and active standing upright because we did not measure CVP in Experiments 1 and 2. However, we have shown in previous work that the decrease of CVP caused by the loss of 1 L of blood is similar to that caused by a lower body negative pressure of 30 mm Hg (Johnson et al., 2014). Other workers have reported that 60° HUTT causes a degree of central hypovolemia, indexed by the decrease in left ventricular end-diastolic volume, that lies between those caused by lower body negative pressures of 20 and 40 mm Hg (Kitano et al., 2005). Taken together, these two pieces of evidence suggest that the loss of 1 L of blood in the supine position in Experiment 3 caused a degree of central hypovolemia comparable to that caused by 65° HUTT in Experiment 1. We cannot exclude that further blood loss in excess of 1 L would have increased SAP-LFSD to the levels observed during 65° HUTT. Nonetheless, arterial baroreflex resonance appears insufficient to explain the whole of our findings on SAP-LFSD. In addition to the cardiopulmonary and arterial baroreflexes, and at variance with blood loss or lower body negative pressure in the supine position, the upright posture entails the stimulation of different proprioceptive and vestibular receptors. At least the activation of vestibular receptors may contribute to the hemodynamic compensation of the upright posture, because vestibular receptors modulate sympathetic activity (Yates et al., 2014), and may interact additively with the arterial baroreflex (Ray, 2000). It is thus conceivable that the increase of SAP-LFSD in the upright position results, in part, from the vestibular modulation of sympathetic activity. Interestingly, the increase in vascular resistance caused by central hypovolemia is greater in the lower than in the upper limbs during HUTT, whereas it does not differ between limbs during lower body negative pressure in the supine position (Kitano et al., 2005). These results suggest that the control of limb vascular resistance exerted by the cardiopulmonary and arterial baroreflexes is modulated by local mechanisms induced by gravitational effects (Kitano et al., 2005). Local vascular factors in skeletal muscles may also impact on SAP-LFSD, because spontaneous arteriolar vasomotion occurs in the LF range (Bertuglia et al., 1996) and is affected by perfusion pressure (Meyer et al., 1988) and tissue metabolism (Pradhan and Chakravarthy, 2011), both of which may change in the leg skeletal muscles in the upright position.

### The enhanced arterial baroreflex control of HP in the upright position may reflect buffering of enhanced fluctuations of vascular resistance

Our results support a direct link between the SAP variability in the LF range and the prevalence of cardiac baroreflex control during wakefulness, as indexed by SAP-LFSD and pCC, respectively, both in the responder subjects of Experiment 1 during HUTT (Figures 2D,E) and in the subjects of Experiment 2 in the seated and standing positions (Figures 4A,B). In stark contrast, the increase in pCC during SWS compared to wakefulness in the supine position was accompanied by a significant decrease in SAP-LFSD (Figures 4A,B). These findings can be interpreted in the light of the emergent properties of a mathematical model of human cardiovascular coupling (Silvani et al., 2011): the arterial baroreflex coupling between HP and SAP fluctuations is enhanced by arterial baroreflex buffering of the SAP changes elicited by vascular resistance fluctuations, and is opposed by central autonomic commands acting on the heart. In this light, the enhancement of the arterial baroreflex coupling between HP and SAP, which we inferred in the responder subjects of Experiment 1 during HUTT and in the subjects of Experiment 2 in the seated and standing positions, may have resulted, at least in part, from an increased variability of vascular resistance. Specularly, the lack of increase of pCC in the non-responder subjects of Experiment 1 during HUTT and in the subjects of Experiment 3 after blood loss may have been, at least in part, because SAP-LFSD did not increase enough. On the other hand, the increase in pCC in subjects lying supine during SWS compared to wakefulness may have occurred despite a decrease in vascular resistance fluctuations because of weaker central autonomic commands to the heart associated with the lack of active engagement with the external environment.

## Conclusions

Our data indicate that upright posture strengthens the coupling between HP and SAP consistently with a greater contribution of the arterial baroreflex to cardiac control. This strengthening can be readily assessed with the CCF analysis, a relatively simple time-domain technique, and occurs in the face of a reduced cBRS. Our results further suggest that the enhanced arterial baroreflex coupling between HP and SAP in the upright position is not triggered by decreased CVP, but rather reflects buffering of enhanced fluctuations of vascular resistance, whereas the decreased cBRS represents a byproduct of decreased modulation of cardiac vagal activity triggered by arterial baroreflex activation.

## Author contributions

AS, BJ, MJ, and PC conceived and designed research; GC, BJ, NvH, GB, AC, MJ, and PC performed experiments and recordings; AS analyzed data, interpreted results of experiments, prepared figures, and drafted manuscript; GC, BJ, NvH, MJ, and PC edited and revised manuscript; all authors approved final version of the manuscript.

## Funding

Support for this study was provided by U.S. Army MRMC Combat Casualty Care Research Program Grant W81XWH-11–1-0823 and American Heart Association Midwest Affiliate Grant 13POST-14380027 to BJ.

### Conflict of interest statement

The authors declare that the research was conducted in the absence of any commercial or financial relationships that could be construed as a potential conflict of interest.
